# Off the charts: A comparative analysis of data visualisations in online science news from South Africa and the USA

**DOI:** 10.1371/journal.pone.0316194

**Published:** 2025-02-21

**Authors:** Marnell Kirsten, Marina Joubert, Ionica Smeets, Winnifred Wijnker

**Affiliations:** 1 Department of Social Sciences, Technology and Arts, Luleå University of Technology, Luleå, Sweden; 2 Centre for Research on Evaluation, Science and Technology (CREST), Stellenbosch University, Stellenbosch, South Africa; 3 Instituut Biologie Leiden, Leiden University, Leiden, Netherlands; 4 Human Experience & Media Design and Quality Journalism in Digital Transition, University of Applied Sciences Utrecht, Utrecht, Netherlands; Wilfrid Laurier University, CANADA

## Abstract

Terms like ‘big data’, ‘data science’, and ‘data visualisation’ have become buzzwords in recent years and are increasingly intertwined with journalism. Data visualisation may further blur the lines between science communication and graphic design. Our study is situated in these overlaps to compare the design of data visualisations in science news stories across four online news media platforms in South Africa and the United States. Our study contributes to an understanding of how well-considered data visualisations are tools for effective storytelling, and offers practical recommendations for using data visualisation in science communication efforts.

## Introduction

Data journalism harnesses the power of data visualisations to help people make sense of large volumes of data and has therefore emerged as a bridge between the explosive growth of data and civil society [1, 2]. Data visualisations have become a common feature of news media and are acknowledged as a key driver of news stories, sometimes even more central than writing [3]. These data visualisations are produced by multi-skilled teams that include journalists, designers, and programmers, as well as cartographers, sociologists, statisticians, mathematicians, economists, urban planners, and more [4].

Data visualisation can simply be defined as the visual representation of data in charts and graphs [5], aiming to facilitate understanding [6]. It combines numeric data with visual representations and design elements [7] and is therefore a blend of science and design that demands a balancing act between accuracy and visual appeal [8].

This present study set out to investigate the data visualisations in science-related news via online news platforms, focused on stories from South Africa and the USA. In the USA, data visualisations in news stories have an established history that has been researched extensively [9–12]. In South Africa, however, this is a particularly new field of scholarship, particularly in science communication and science journalism. Therefore, we compared data visualisations from South Africa and the USA to help fill this knowledge gap.

## Theoretical framework

Rose [13] shows that when researchers study visual texts, like data visualisations, there are three possible sites of focus, namely the site of production, the site of the image, and the site of audiencing [13]. Our study is predominantly located at the site of the image, focusing on the design elements, purpose or intent, and the kind of data stories told by data visualisations. This results in an interdisciplinary study in which graphic design and science communication are equally important considerations in the understanding of how data visualisations are used in science news stories. We make recommendations for future studies located at the site of production and the site of audiencing. The three sites that Rose identifies are not entirely distinct from each other and an understanding of one may aid an understanding of the other two. For this reason, this framework will outline important implications related to data visualisation in news stories as relevant to the site of production [the purpose of data visualisations in news stories] and the site of audiencing [graph literacy and cultural specificity], as they are relevant in answering our research questions. Thereafter, this framework focuses on the site of the image itself, outlining the theoretical foundation of the codebook we used in this study.

### The site of production: The purpose of data visualisations in news stories

The most basic function of data visualisations relates to its ability to synthesise communication on a specific topic and provide a new way to see the data [4]. Therefore, effective data visualisations in news stories can be powerful tools that facilitate concise communication of complex information and help readers to interpret the information behind a news story, thereby enhancing their understanding of a specific issue [14, 15].

The ability of data visualisations to attract attention may be linked to its potential to humanise data, incorporate empathy, and help audiences to connect to information [7, 16], thereby facilitating a connection with the content of the news story. A study by Kennedy and Hill [17] reports that data visualisations encourage people to reflect on how they feel about an issue, adding that people experience a wide range of emotions when they engage with data visualisations, including pleasure, anger, sadness, guilt, shame, relief, worry, love, empathy, excitement, and offence. Such affective influences may be strengthened by the aesthetic appeal of a visualisation [1].

There are therefore implications for the look and feel of a data visualisation based on the purpose or the intention of the data visualisation in the news story. Ojo and Heravi [18] develop a typology of the purpose or intent of data visualisations in the context of award-winning science news stories that can help scholars better understand the connection between journalism and data visualisation. These authors identify the follow purposes or intentions: 1) to refute claims, 2) to reveal unintended consequences, 3) to reveal information of personal or public interest, 4) to enable a deeper understanding of a certain phenomenon, 5) to reveal anomalies and deficiencies in systems, 6) to track changes in systems, and 7) to reveal information about an entity in increasing levels of detail [18].

Supplementing the above typology, Cohen [19] identifies some of the main types of data stories that visualisations can help to tell. These are to show changes over time, to compare values, to highlight connections between variables, to trace flows, to show hierarchy, to browse large databases, and to envision alternate outcomes along with actual situations or figures. Cohen writes that data visualisation “offers the tantalising opportunity for storytelling that is above all driven by facts, not fanaticism” [19].

### The site of audiencing: Graph literacy and cultural specificity

Data visualisations are novel forms of news content that make new demands on those who create it, but also on those at the receiving end. The creation of data visualisations involves a process of coding to represent the data in a specific way. Readers have to decode these visualisations through a process of visual perception and cognitive processing in order to interpret and understand it [4]. Meaningful interpretation of data visualisations may require skills such as language skills, mathematical and statistical skills, or critical thinking skills [5, 17]. The ability of people to read graphs closely and accurately so that they can decode and interpret the information presented in a data visualisation is known as ‘graph literacy’ [14]. It is a skill that builds on literacy, numeracy, and visual processing and can also be encountered in other literature as ‘visual-numeric literacy’ [20] or ‘graphicacy’ [21]. Importantly, people will only engage with data visualisations if they feel confident about their ability to understand the visualisations [22].

Different types of data visualisations may demand different levels of skills and amounts of time to interpret. For example, it may be easier for people to interpret simple bar and line charts compared to complex data visualisations such as scatterplots, even though the latter may be more aesthetically pleasing. Another important factor that makes the interpretation and understanding of data visualisations more complicated, is the arbitrary and conventional nature of language systems–this includes visual language [23]. A stable meaning and interpretation of language, including data visualisation as a language, relies on a culturally agreed upon relationship between what something shows or visualises and what it actually means. The meaning of data visualisations, including its use of colour, representation of chronology, etc., may therefore be culturally or linguistically specific and may not be grasped by its entire audience.

### The site of the image: Seven important themes in understanding design elements of data visualisations used in science news stories

In *Storytelling with Data*, Knaflic [2] shows that design considerations can help a data visualisation practitioner to tell compelling stories–this includes a storytelling function in the context of science news stories. Knaflic shows that when a data visualisation has a well-considered design, with the audience and their understanding of the data visualisation in mind, it has the potential to tell powerful and effective stories. Cohen [19] emphasises Knaflic’s contention, writing that “a well-designed data visualisation can give viewers an immediate and profound impression, and cut through the clutter of a complex story to get right to the point”.

Based on the work of Knaflic [2], Tufte [24], Wainer [25], Wilke [26], Ojo and Heravi [18], and Cohen [19], we formulated seven themes for evaluating the use of data visualisations in online science news stories, namely:

1. **The type of visualization**
  Knaflic shows that “in many cases, there isn’t a single correct visual display; but rather different types of visuals that could meet a given need” [2]. It is crucial to understand which kind of data visualisation will optimally complement the news story.
2. **The use of a title, subtitles, and provision of data sources**
  This theme relates to Knaflic’s emphasis on the importance of context [2]. When research is focused on the site of the image, the title and a subtitle can be used as a proxy for providing the reader with a certain level of context.
3. **The use of colour**
  Wilke [26] shows that colour in data visualisation can be used in the following three ways: “We can use colour to distinguish groups of data from each other, to represent data values, and to highlight” [26]. It is therefore important to consider colour combinations, but also how colour is used for visual emphasis (theme six).
4. **The use of text and labels**
  Knaflic writes that a “thoughtful use of text helps ensure that your data visualisation is accessible. Text plays a number of roles in communicating with data: use it to label, introduce, explain, reinforce, highlight, recommend, and tell a story” [2]. This includes the use of titles and subtitles, labelling specific elements of the data visualisations, explaining the relationship between different components of the data visualisations, and including legends or axes labels, where relevant.
5. **The use of additional visual elements**
  This theme includes the use of basic visual elements (lines, arrows, dots), the use of simple or detailed additional illustrations, and the use of any other additional visual elements (a photograph, a second visualisation, a map, a graphic element, etc.) in the data visualisation. The use of additional visual elements is ideally not adding “visual clutter [that] creates excessive cognitive load that can hinder the transmission of [the] message” [2].
6. **The use of visual emphasis**
  Knaflic shows that this is important, as “without other cues, our audience is left to process all of the information we put in front of them” [2]. It is important to consider the data visualisation’s use of text, colour, scaling, and a change in the look and feel of lines as a way to add visual emphasis.
7. **Purpose and intent of the visualisation in the news story**
  This theme overlaps with the site of production, and the aforementioned typologies by Ojo and Heravi [18] and Cohen [19] are useful to make sense of this aspect of the use of data visualisations in online science news stories. We focus on: a) the topics of the science news stories, b) the purpose or intent of the data visualisations in the news stories, c) the kind of data stories that the data visualisations told in the context of the news story.

The purpose of critical reflection on data visualisations can be to understand what works [or not] for different audiences and on different topics (site of audiencing), and to advance the responsible and ethical use of data visualisations as a journalistic tool (site of production). Visualisations may attract criticism, including poor design, being crowded and confusing, or being beautiful but unclear [27]. Schwabish [27] classifies prominent criticisms of data visualisation to fall either into the “GraphCrimes” camp or the “Xenographics” camp. The first refers to a tendency to judge data visualisation on binary value as either ‘good’ or ‘bad’, but also potentially providing valuable feedback, while xenographics refers to criticising data visualisations that do not follow existing guidelines and are not visually pleasing [27]. Our study relies on the useful guidelines that Knaflic sets up, as explored in the seven themes that underpin our codebook. However, aligning with Schwabish’s call for fewer binary value judgments about certain data visualisation types as solely ‘good’ or ‘bad’, we focus on the potential of design elements of data visualisations to contribute to effective storytelling. For example, we use pie charts to present our own findings, even though Knaflic feels pie charts are “evil” [2]. In this way we hope to contribute to constructive discussions around the effective use of data visualisations.

## Study objective and research questions

The present study set out to compare data visualisations in science-related news stories via online news platforms in South Africa and the USA. We aimed to combine practical guidelines for creating data visualisations, supported by academic reflections on data visualisation in order to contribute to the body of knowledge about data visualisations and its links with science communication. In particular, we wanted to contribute to science communication scholarship in and from Africa, a region that is underrepresented in this type of research [28]. A 2022 study by Wijnker and colleagues tests “four correction methods as debunking strategies to correct bar charts with manipulated vertical axes” [29] in a US context. Building on this work, arguably situated in Rose’s site of audiencing [13], we aimed to get a better understanding of the site of the image in a US context. Furthermore, we wanted to compare this US ‘site of image’ with that of South Africa, in order to help fill the aforementioned knowledge gap on journalistic science communication in South African and on the African continent.

We compared the design elements of data visualisations and their relationship to the content of the news stories, and explored the visual and textual elements of data visualisations to understand how these may contribute to or detract from the content and understanding of online science news stories. We used the following two research questions to guide our study:

RQ1: What are the characteristics of data visualisations in South Africa and in the USA in terms of their design choices (related to the type of visualisation, use of a title, text, colour, additional visual elements and visual emphasis) and how does this compare between the two countries?
RQ2: How is the content of the science news stories related to the design of data visualisations and how does this compare between the two countries?

## Methodology

The current study employed qualitative content analysis as its research approach, following the seven steps of this analytical process as suggested by Hsieh and Shannon [30]. We made use of quantitative methods and statistical approaches to refine our codebook and test for coder reliability–this is elaborated on in following subsections.

### Sample selection

We collected data from four online English language news platforms, two each in South Africa and the USA, published from 1–31 October 2022. We selected this timeframe to minimise the influence of the Covid-19 pandemic on our results, and to work in a timeframe that was an active academic period for both countries outside of major public holidays. The period also coincided month of the Nobel Prize giving, meaning that we could expect to find news from research institutions from both countries during this period. We were restricted to only one month of data collection based on budgetary constraints, but we also reached saturation in terms of the diversity of data stories by the end of October, meaning that new data were no longer showing any great differences or diversity.

Since we were interested in science news, we searched for articles that contained one or more keywords that have been confirmed by an earlier study [31] as a way to identify science-related content in online news media. These keywords are: “scien*, stud*, result*, research*, universit* and institut*” [31]. In the articles with these keywords, we searched for stories that contained one or more data visualisations. We downloaded all relevant stories.

We included stories that visualised quantitative or qualitative information or both. The data visualisations could be static, interactive (changing in response to active intervention by the reader) or dynamic (visualizing changes over time without requiring the active intervention of the reader). We included infographics and maps, provided they contained a visualisation of quantitative data relevant to the news story.

All examples that contained no colour (i.e., data visualisations in black and white or grayscale) and no text (this includes no annotations, labelling, titles, etc.) were removed from the dataset. We only analysed one visualisation per article, selecting either the main (most prominent) visualisation or the first visualisation in each article. Furthermore, duplicate examples were removed.

**Fig 1:**
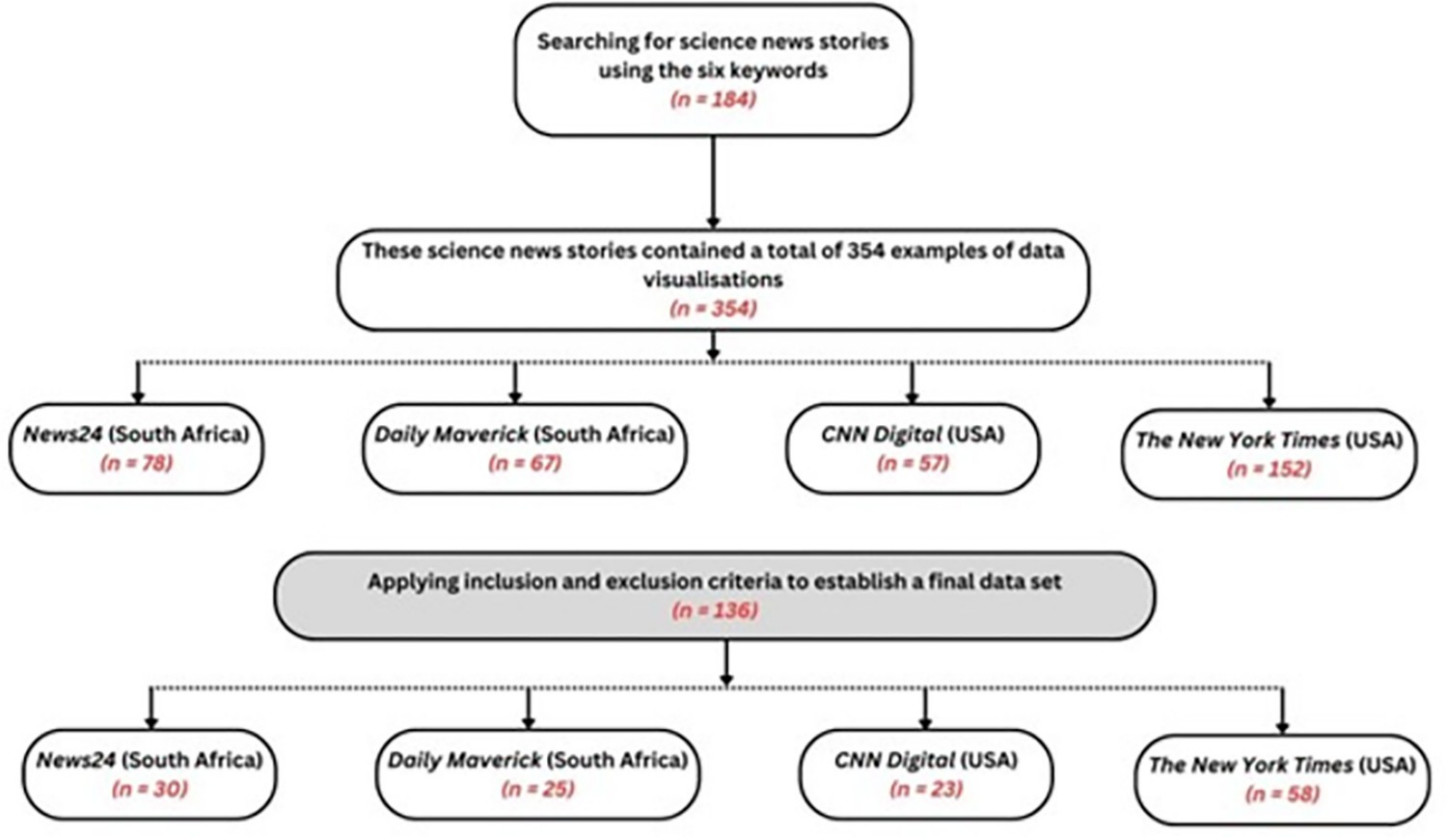
Diagram of search results and process.

Our search strategy yielded 184 science news stories containing 354 examples of data visualisations. After applying the inclusion and exclusion criteria listed above, the dataset was reduced to 136 data visualisations for further analysis. Fig 1 shows a schematic representation of these results.

### Defining categories

We based our codebook on the seven themes as presented in the theoretical framework, being: 1) type of visualization; 2) the use of a title, subtitles, and provision of data sources; 3) the use of colour; 4) the use of text and labels; 5) the use of additional visual elements; 6) the use of visual emphasis; and 7) purpose and intent of the visualisation in the news story, and therefore followed the parameters of Hsieh and Shannon’s description of a directed content analysis [30]. A complete breakdown of how these themes were defined and coded can be seen in our codebook.

### Coding–Process, implementation and reliability

At the start of the project, two coders collaborated to develop, test, and refine the codebook with a preliminary dataset. We applied Cohen’s Kappa to assess the intercoder reliability during this process to ensure that the final codebook was as refined and sound as possible. Following this test run and refinement, the first author (a scholar in the field of data visualisation and design) coded the full final dataset. The intercoder reliability of this initial step had no bearing on the intra-coder reliability calculated for the complete final dataset.

For the complete final dataset, unweighted Cohen’s Kappa was calculated to test the intra-coder reliability, based on the same 10% of the data coded twice, three weeks apart. Kappa was not calculated for descriptive variables such as date of publication and country of publication, leaving 38 variables. Of these, Kappa could not be calculated for six variables because either no values were recorded or all observations within the variable were the same across both time points. For the latter, perfect intra-rater agreement can be assumed. For the remaining 32 variables, the Kappa ranged from .53 to 1. Only one variable had moderate agreement, seven had substantial agreement, and the remainder had near-perfect agreement.

### Analysis

**Fig 2:**
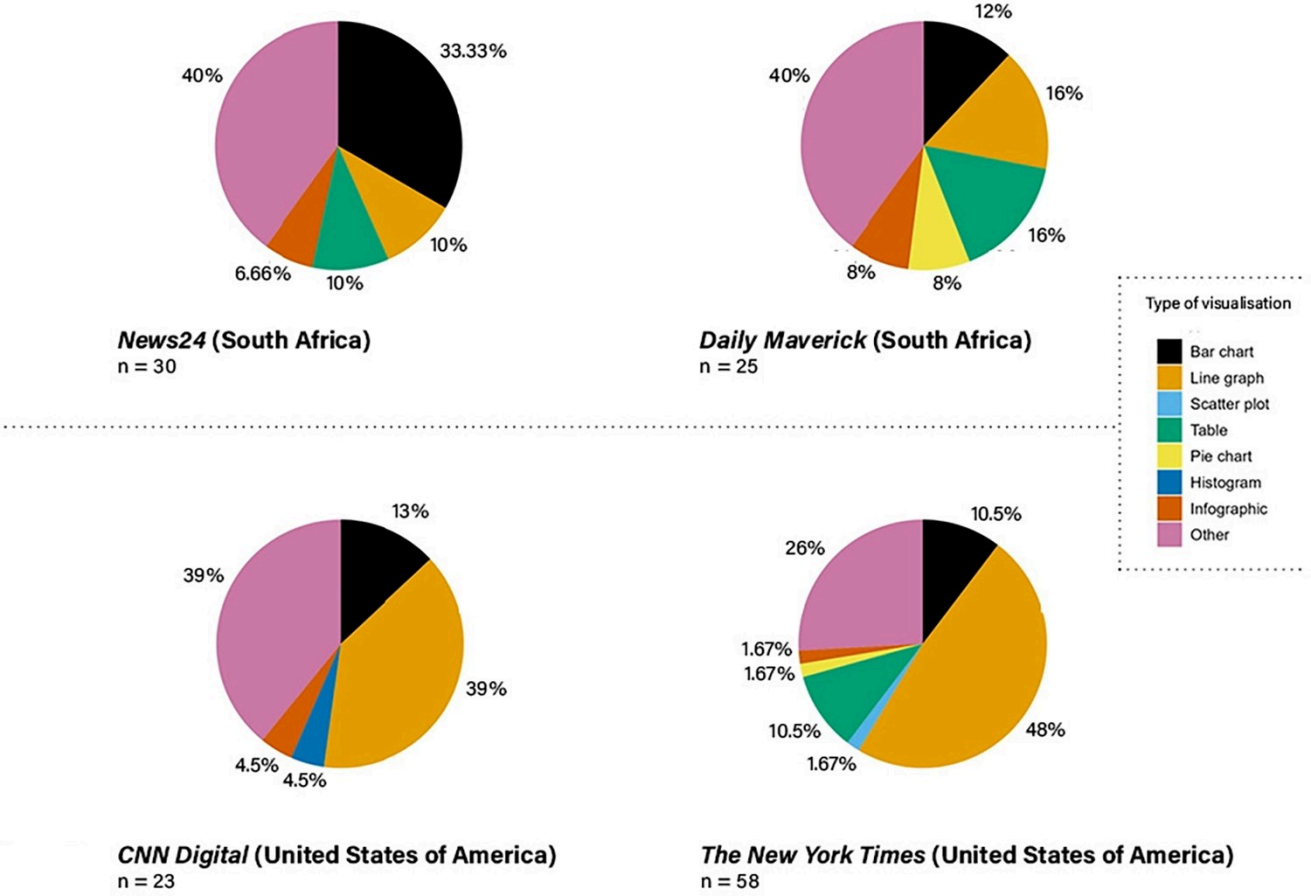
Type of data visualisations used per platform (colour scheme selected for increased reader accessibility).

After coding the data, quantitative analysis was done on JASP, a free and open-source program for statistical analysis. This analysis yielded the figures that our results and discussion section refers to throughout, and helped with the basic visualisations that underpin Figs 2 and 14. The qualitative analysis of this study links the quantitative results back to the sources and authors explored in the theoretical framework.

## Results and discussion

In our dataset of news stories that contained data visualisations (N = 136), most were in *The New York Times* (n = 58), followed by *News24* (n = 30), *Daily Maverick* (n = 25) and *CNN Digital* (n = 23)–see Fig 1. Below, we discuss our findings, and present some illustrative examples, using the seven themes of our codebook to provide structure. As explained before, the discussion focuses predominantly on the site of the image itself, while our conclusions and recommendations for future studies, also consider the site of production and the site of audiencing.

### Theme 1: Type of visualisation

From the ‘conventional’ or more commonly used data visualisations, bar charts were most often used in *News24* stories (10 examples) and in stories on *Daily Maverick*, line graphs (4 examples) and tables (4 examples) featured most–see Table 1. However, mostly occurring on both these platforms were ‘other’ data visualisations. For *Daily Maverick*, these ‘other’ data visualisations were mostly stacked bar charts (3 examples) and pareto charts (2 examples), while *News24* mostly made use of control charts (3 examples), grouped bar charts (2 examples) and scaled-up numbers (2 examples).

**Table 1:** Types of data visualisation per platform.

	*News24*	*Daily Maverick*	*CNN Digital*	*The New York Times*	Total
Bar chart	10	3	3	6	**22**
Line graph	3	4	9	27	**43**
Scatter plot	-	-	-	1	**1**
Table	3	4	-	6	**13**
Pie chart	-	2	-	1	**3**
Histogram	-	-	1	-	**1**
Density plot	-	-	-	-	**-**
Infographic	2	2	1	1	**6**
Other	12	10	9	16	**47**

From the list of ‘conventional’ or commonly used data visualisations, line graphs were most often used in science news stories on *CNN Digital* (9 examples) and on *The New York Times* (27 examples). On both these platforms the use of ‘other’ data visualisations was also common– 9 on *CNN Digital* and 15 on *The New York Times*. On *CNN Digital*, these ‘other’ data visualisations were mostly isoline maps (4 examples) and choropleth maps (3 examples), while on *The New York Times*, stacked bar charts were used most frequently (5 examples). The types of data visualisations (except for ‘other’) with the highest frequency are indicated with grey shading.

We see that (Fig 2) bar charts and line graphs were used by all four platforms, and jointly accounted for more than half of all data visualisations in the US stories, confirming that these basic and well-known visualisations remain popular.

Some news stories from the USA contained more complex types of visualisations, such as histograms and scatter plots, that were not found in the South African data. The widespread use of ‘other’ data visualisations, and the variety of data visualisations in this grouping, in stories on *The New York Times*, in comparison to those on the other three platforms, relates to Knight’s contention [32] that elite mainstream media use more complex visualizations compared to tabloid press, as explored in the theoretical framework.

### Theme 2: The use of a title, subtitles, and provision of data sources

Table 2 summarises the use of titles, subtitles, and the disclosure of data sources across the four news platforms. Titles and subtitles had to be clearly distinguishable from each other, and the subtitle could not be a legend or a source of data. Sources of data had to be explicitly indicated as such.

**Table 2:** The use of titles, subtitles and disclosure of data sources in data visualisations across the four platforms.

		*News24*	*Daily Maverick*	*CNN Digital*	*The New York Times*
Does the data visualisation have a **title**?	Yes	28	23	21	56
	No	2	2	2	2
Does the data visualisation have a **subtitle** that adds context or information to either the title or the data visualisation itself?	Yes	13	13	18	20
	No	17	12	5	38
Does the data visualisation specify a **source of the data**, either in the title, a footer, or anywhere else in the data visualisation?	Yes	20	18	22	55
	No	10	7	1	3

As can be seen in the above table, across all four platforms the use of a title for data visualisations was very widespread, while the use of subtitles was most prevalent on *CNN Digital*. Sources of data are mostly disclosed across all four platforms. These three elements of additional information given to the data visualisation audiences help them make sense of the data visualisation. The disclosure of a source of data helps to solidify the information conveyed as accurate and reliable, even though this does not guarantee the actual visualisation of the data to be accurate and accessible. Types of sources may also impact the credibility of the underlying data [33].

### Theme 3: The use of colour

In data visualisations in science news stories on *News24*, the combination of two to three colours were most often used (14 instances), followed by the use of three or more colours (10 instances) and the use of single colour (6 instances). Within these combinations, a distinct qualitative colour scale was mostly used (16 instances), followed by the use of a similar qualitative colour scale (6 instances). A sequential colour scale was used in only two data visualisations on this platform. Data visualisations on *Daily Maverick* also mostly used a combination of two or three colours (13 instances), followed by the use of three or more colours (8 instances), and the use of single colour (4 instances). Using a distinct qualitative colour scale was against the most prominent (16 instances), followed by the use of a similar qualitative colour scale (5 instances). The use of single colour was limited to four data visualisations on this platform.

The gap in frequency between the use of single colour and multiple colours in data visualisations is not quite as wide on the US platforms as on the South African news platforms. *CNN Digital* made most use of single colour (9 instances), followed by combinations of two or three colours and three of more colours (7 instances each). However, these combinations saw a more diverse use of colours scales than on South African platforms. The use of a sequential colour scheme was most common on *CNN Digital* (6 instances). This was followed by the use of a distinct qualitative colour scale (4 instances) and a similar qualitative colour scale (3 instances). A divergent colour scale was used once on this platform. Data visualisations on *The New York Times* most often made use of a combination of two or three colours (25 instances), followed by the use of single colour (22 instances), and the combination of three or more colours (11 instances). These combinations see the use of a distinct qualitative colour scale as the most common by far (27 instances). This is followed by the use of a similar qualitative colour scale (6 instances), a divergent colour scale (two instances), and one instance of the use of a sequential colour scale.

**Fig 3:**
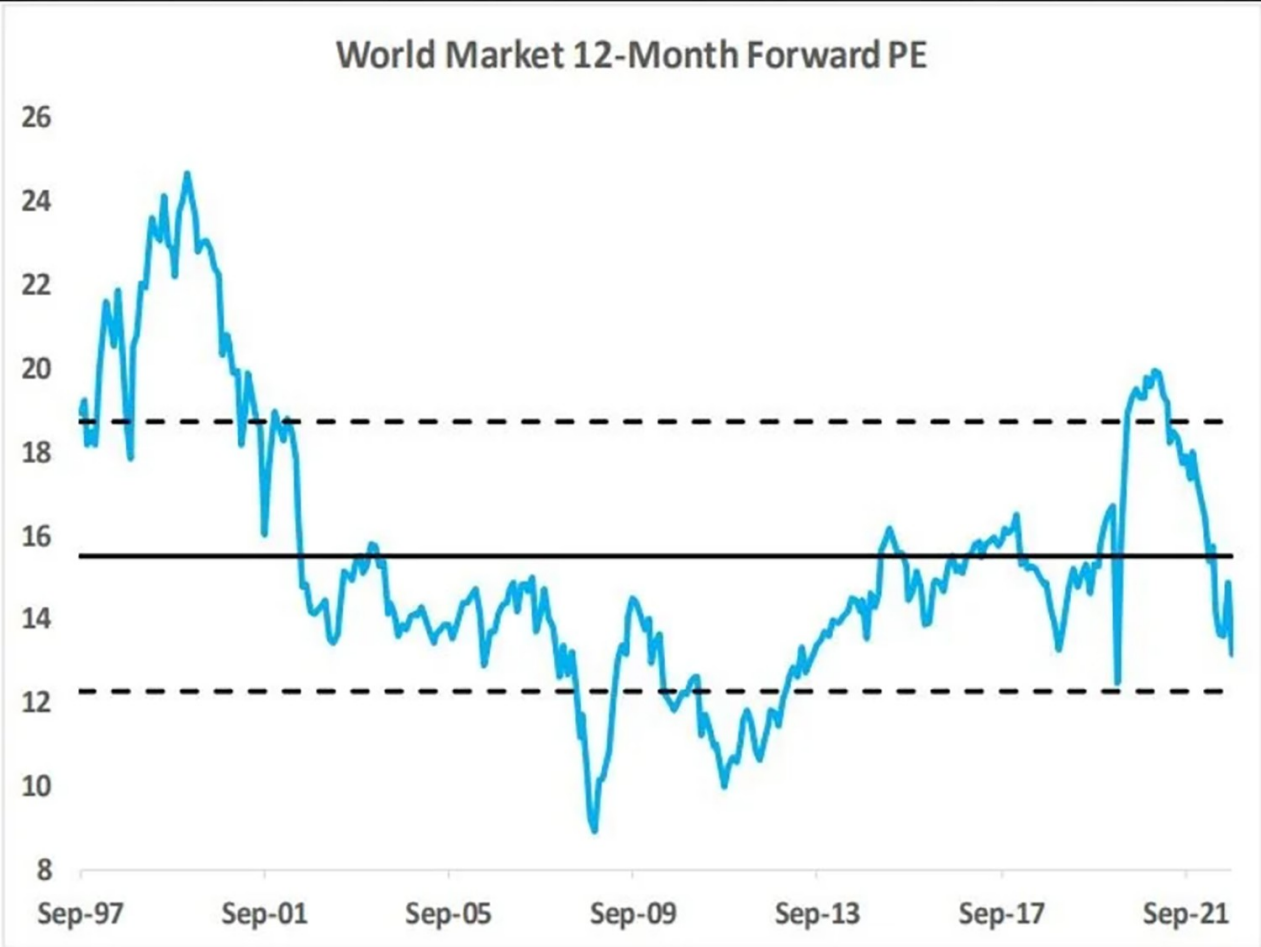
*News24*, ‘World market 12-month forward PE’, published on 3 October 2022.

In data from both countries, we found that using one single colour mostly did not clarify or obstruct the meaning of the data visualisation. For example, the blue line in Fig 3 may help this graph to stand out on a page but does not enhance understanding or clarify the meaning of the chart–it does not hinder meaning either.

**Fig 4:**
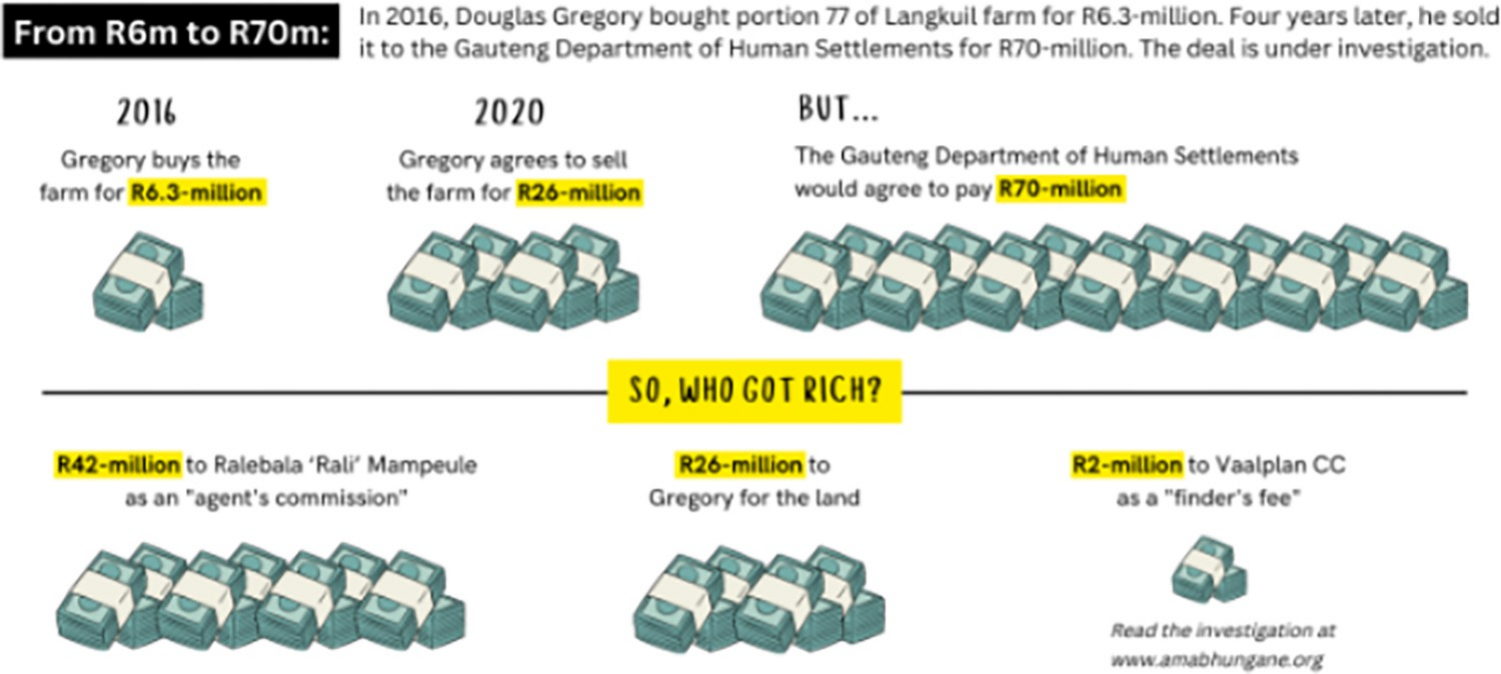
*Daily Maverick*, ‘So, who got rich?’, published on 13 October 2022.

Across all platforms, we found that the use of multiple colours, especially three or more colours, were functional in terms of clarifying meaning and aiding readers’ understanding. For example, in Fig 4, the use of green and beige for the illustrations of the banknotes and their bindings solidifies an understanding of the subject of the visualisation, while the use of yellow text boxes gives the impression of highlighting and draws attention to the amounts that the green banknote illustrations represent.

**Fig 5:**
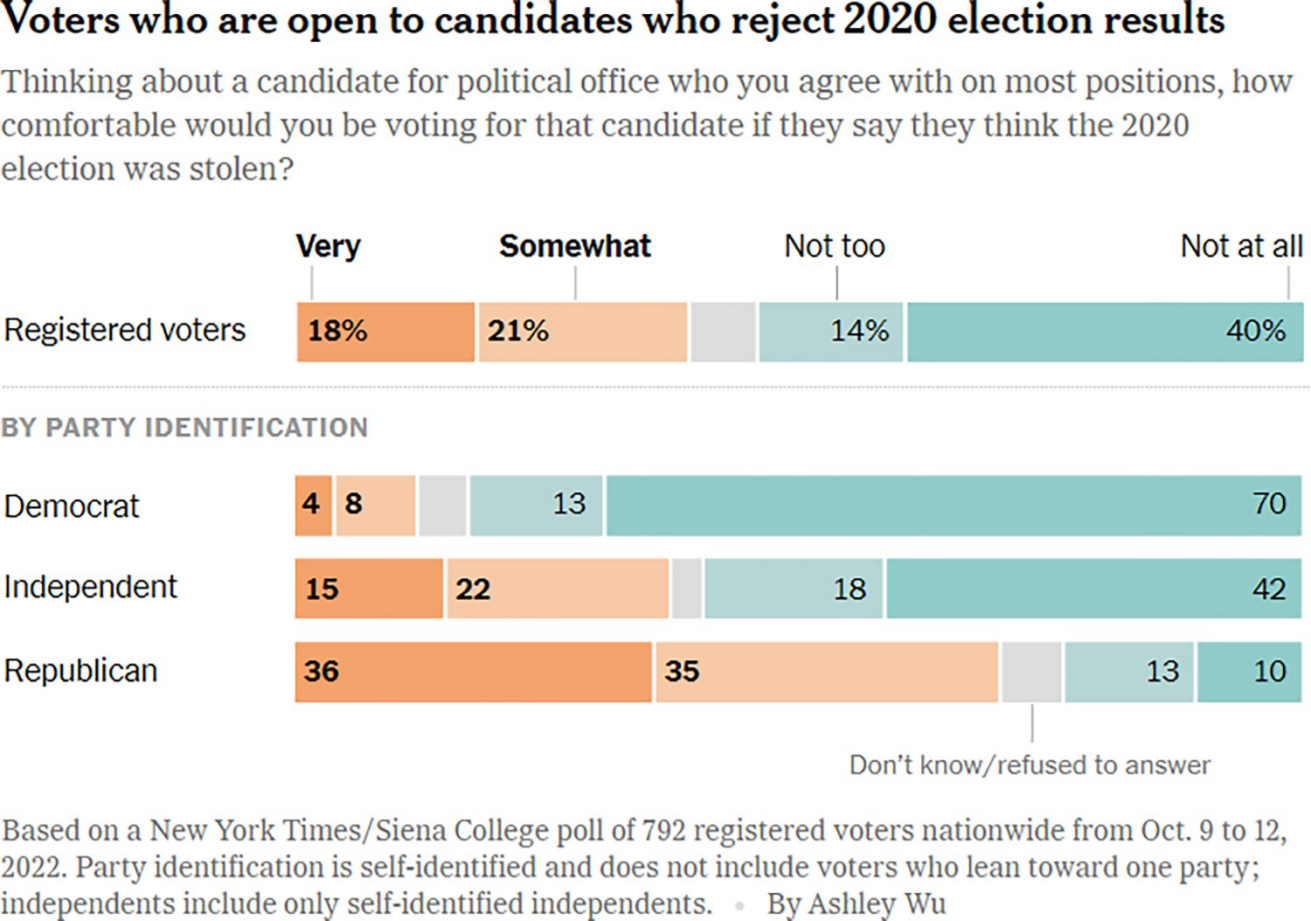
*New York Times*, ‘Voters see democracy in peril, but saving it isn’t a priority’, published on 18 October 2022.

Fig 5 presents a good example of how a divergent colour scale can help to clarify the message of a data visualisation. In this stacked bar chart, a complementary and divergent colour scale is used to draw attention to a range of voter sentiments in the US. The orange and turquoise colours visually read as closely related to Republican red and Democratic blue, helping to ensure that this data visualisation is understood within the context of US politics. At the same time, these colours are distinct from red and blue, and help the reader to interpret these sentiments along the intended ranges of ‘Very’ to ‘Not at all.’

### Theme 4: The use of text and labels

The use of text and labelling in data visualisations across all four platforms is summarised in Table 3 below. The table distinguishes between the use of text to label specific elements in the data visualisation, the use of text to provide additional context to help the visualisation make sense to the reader, and the use of text to explain the relationship/s between different elements in the data visualisation to the reader.

**Table 3:** The use of text in data visualisations across the four platforms.

		*News24*	*Daily Maverick*	*CNN Digital*	*The New York Times*
Text was used to describe **specific elements** in the data visualisation.	Yes	13	14	21	36
	No	17	11	2	22
Text was used to describe the data visualisation as a whole or to **provide context** to the reader.	Yes	14	12	21	26
	No	16	13	2	32
Text was used to **describe the relationship/s** of different elements in the data visualisation to the reader.	Yes	2	4	1	5
	No	28	21	22	53

Across all the platforms, except for *News24*, text was frequently used to describe specific elements of data visualisations. *CNN Digital* was the only platform that mostly used text to provide additional context or to describe the visualisation. None of the four platforms used text to describe the relationship/s of the different elements in the data visualisation.

**Fig 6:**
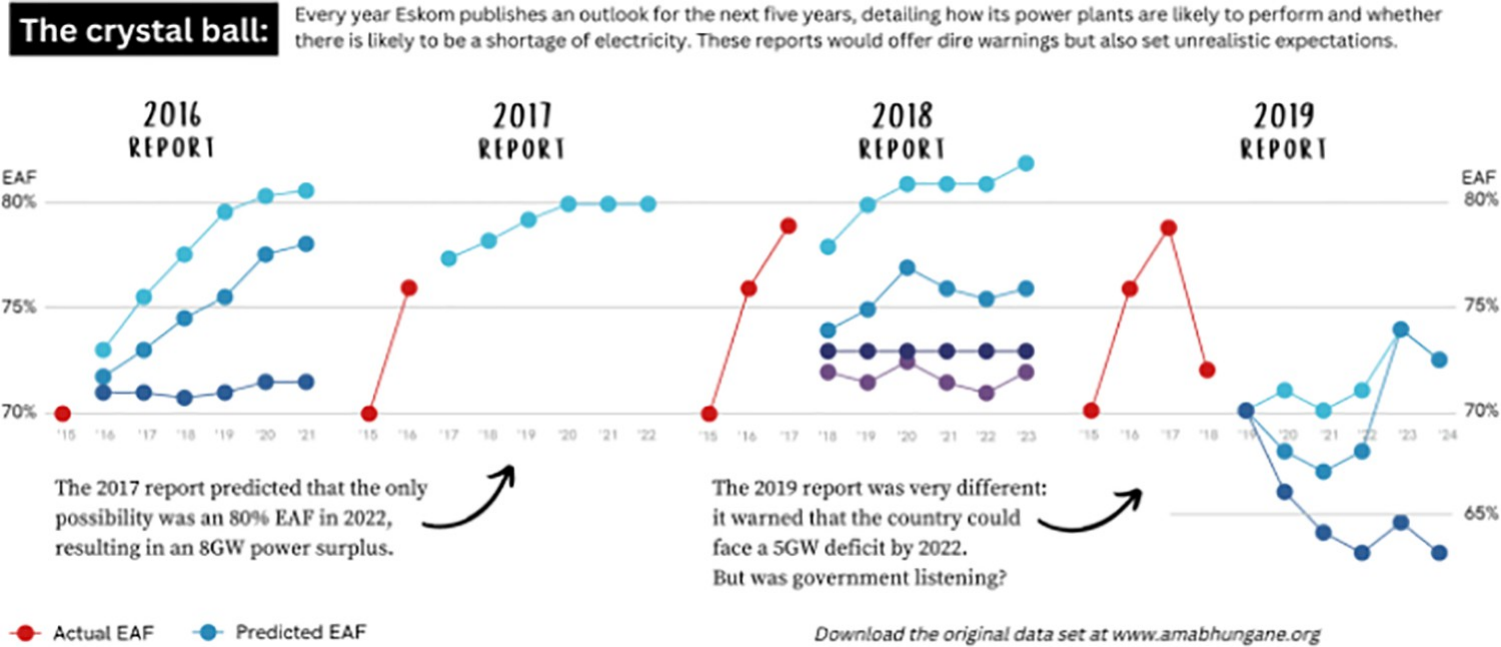
*Daily Maverick*, ‘The crystal ball’, published on 3 October 2022.

Across all four platforms, we found that when text was used to label specific design elements, it helped to clarify the meaning and enhance understanding of visualisations. Of the total 84 data visualisations in our data set that used text to label specific elements, 71 instances saw a use of text in a way that helped to clarify the meaning of the data visualisation (13, 6, 20, and 32 for *News24*, *Daily Maverick*, *CNN Digital*, and *The New York Times*, in that order). For example, in Fig 6, textual components label specific elements in the data visualisation and help the reader to make sense of a busy visualisation by highlighting the relationship between its elements.

**Fig 7:**
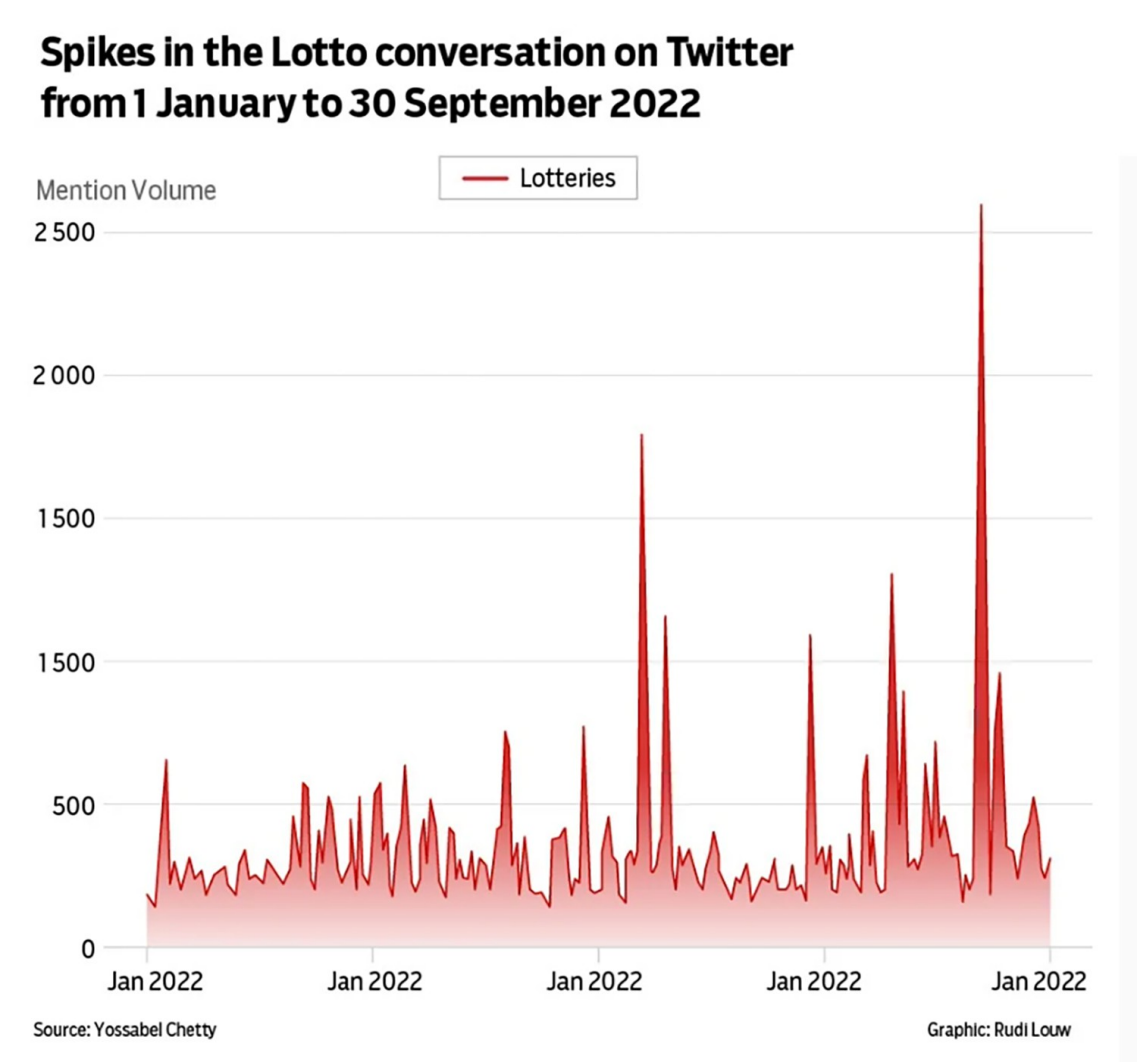
*News24*, ‘Spikes in the Lotto conversation on Twitter from 1 January to 30 September 2022’, published 20 October 2022.

Fig 7 is an example of how text can confuse the reader and obstruct meaning. The labels of the x-axis are incorrect (erroneously, all five read ‘Jan 2022’), and the other textual elements do little to help tell a data story. Knaflic [2] argues that it is unsafe to “assume that two different people looking at the same data visualisation will draw the same conclusion. If there is a conclusion you want your audience to reach, state it in words”. Annotations could have helped the reader understand the peaks in this data visualisation in relation to a wider context and also in relation to the accompanying news story.

**Fig 8:**
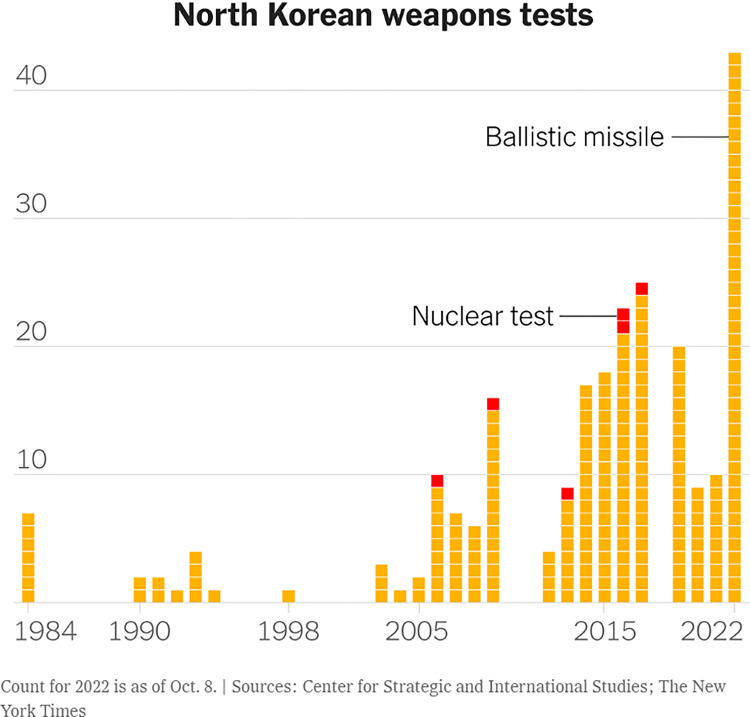
*The New York Times*, ‘North Korean weapons test’, published on 9 October 2022.

In Fig 8, the textual elements are clear and used effectively to annotate and explain what the visualisation is about, including an identification of the data source which supports credibility. In this case, colour furthermore helps to clarify the visualisation with yellow signifying ballistic missile tests and red drawing attention to nuclear tests. The use of red as visually more intense helps the reader to conceptually make sense of the visualisation and read these as explosions. This use of colour is symbolic, and this kind of meaning-making is dependent on shared convention and cultural systems [23]. This highlights another complicated aspect of visual-numeric literacy [20]–the function of culturally specific interpretations of design elements.

**Fig 9:**
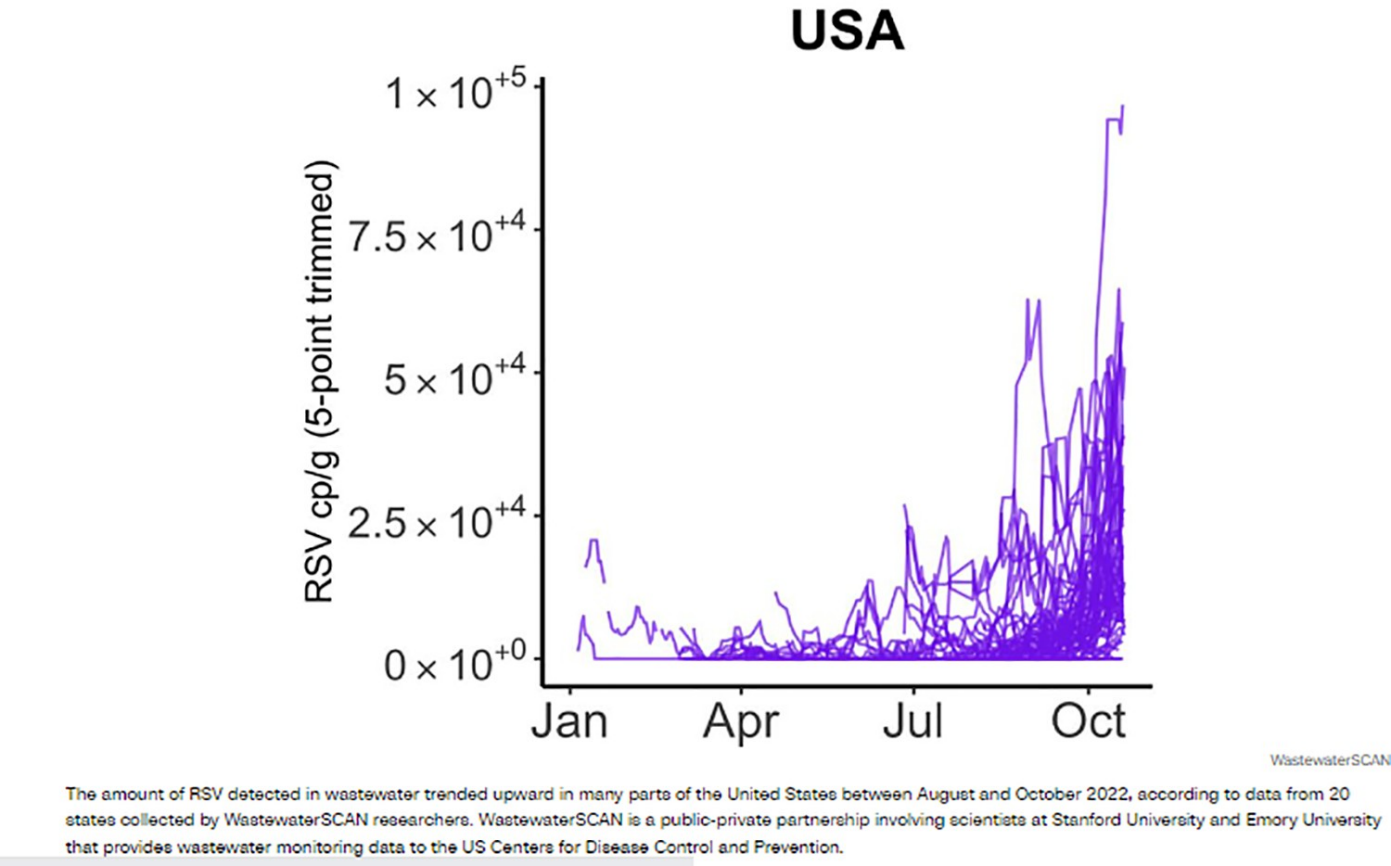
*CNN Digital*, ‘USA’, published on 27 October 2022.

In the case of Fig 9, text is sparse and highly technical, and it is unlikely that the general readership of a platform like *CNN Digital* would be able to make sense of the y-axis labels and text below the visualisation. The use of a single colour and busy composition of the graph itself contribute to a data visualisation that is inaccessible and a data story that is ineffective.

### Theme 5: The use of additional visual elements

In our dataset, 78 out of the overall 136 data visualisations included additional visual elements. On three of the four platforms our study focused on, more than half of the examples included additional visual elements (19, 13, and 36 for *News24*, *CNN Digital*, and *The New York Times*, in that order). Of the 25 data visualisations from *Daily Maverick*, 10 included additional visual elements.

**Fig 10:**
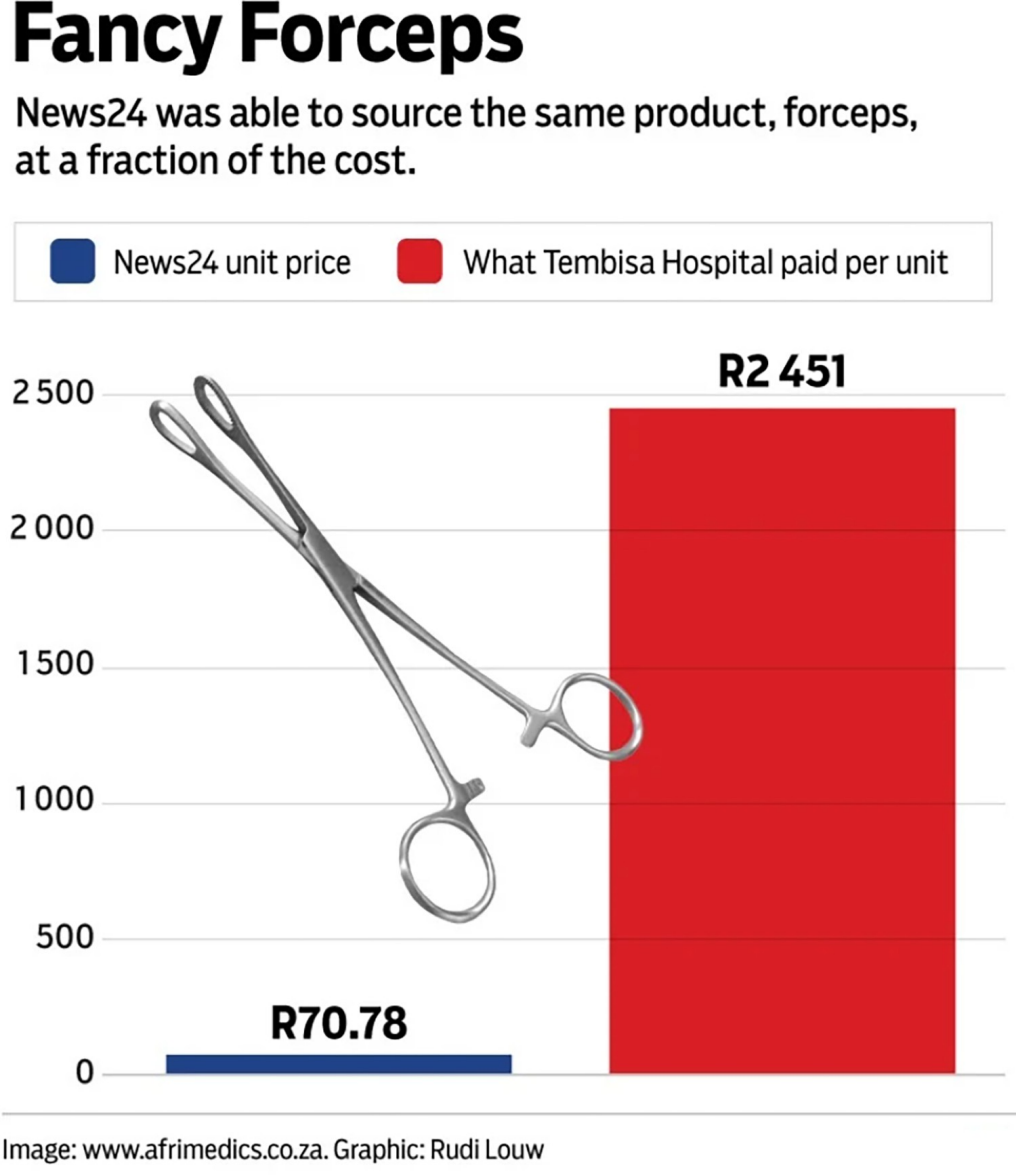
*News24*, ‘Fancy forceps’, published on 7 October 2022.

In the South African data, we found that simple visual elements could help to clarify the meaning of a visualisation (see, for example, the use of arrows in Fig 6). While these arrows may be considered as “distractions” [2], they also solidify a chronology in the data story and help to make the data visualisation accessible to readers with varying degrees of visual-numeric literacy [20]. In another example (Fig 10) the addition of the image of a forceps may serve to attract attention, but it does not contribute to clarify the meaning of the visualisation, and could be considered as ‘visual noise’ [34].

**Fig 11:**
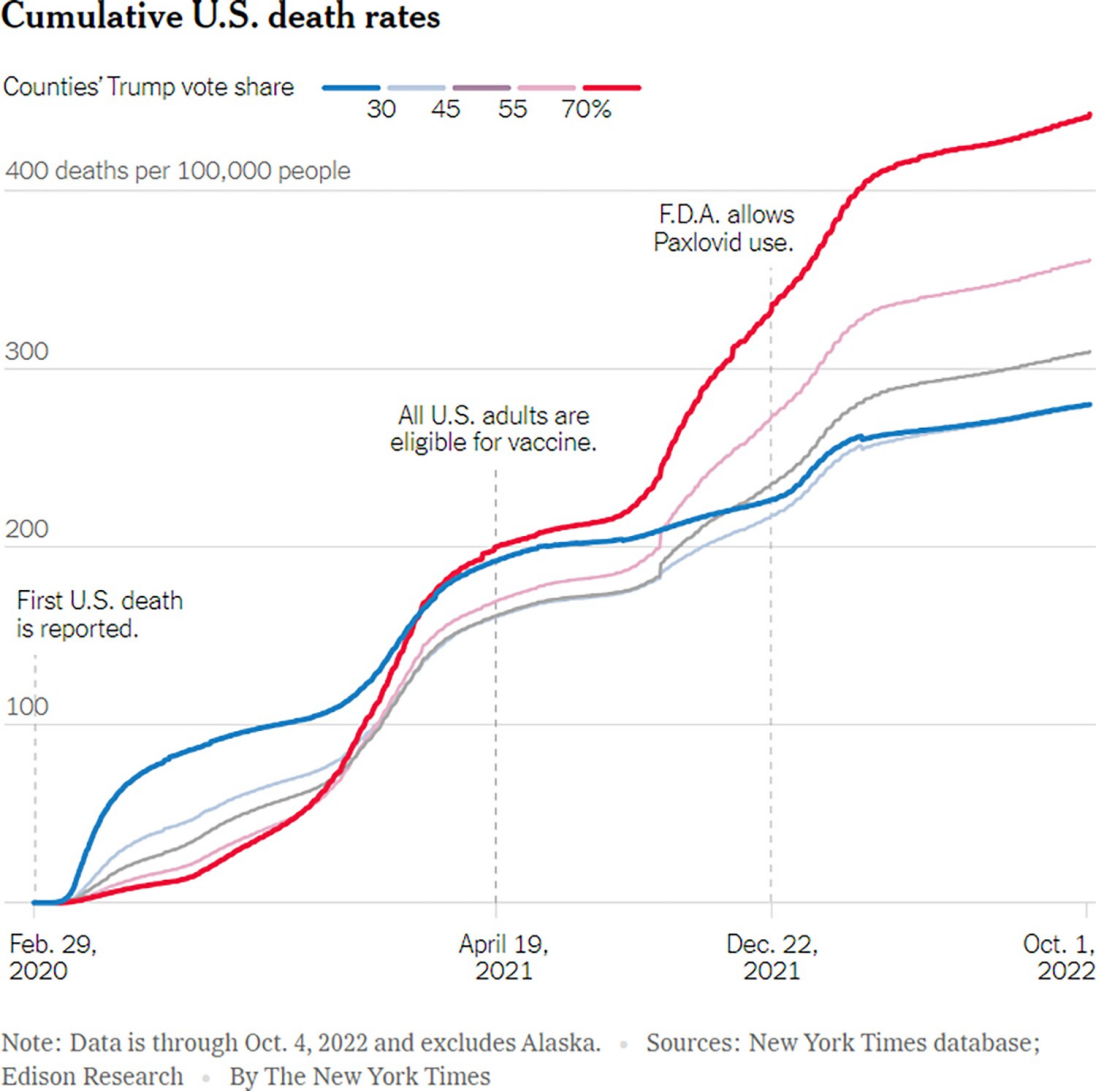
*The New York Times*, ‘Cumulative U.S. death rates’, published on 7 October 2022.

The US platforms did, however, use simple icons (for example arrows), and visual elements such as photos and maps, as well as supplementary data visualisations embedded inside a main visualisation. In most cases, these additional visual elements helped to create a well-rounded data story, offering layers of simplified complexity that make sense in the data visualisation’s system of meaning-making [23]. Fig 11 offers an example of the use of simple visual elements–dotted lines–from *The New York Times*. The lines serve to highlight moments on the timeline that the x-axis represents, using additional text to also contextualise these points in time. Furthermore, the dotted lines also serve as an example of adding visual emphasis to data visualisations.

### Theme 6: The use of visual emphasis

Of the 30 data visualisations from *News24*, 27 made use of some sort of visual emphasis to draw the reader’s attention to important or interesting details. Twenty-one made use of visual emphasis by some effect to the text (boldened text, italics, difference in font size, some aspects of text in a different colour–like highlighting), seven used different colour tones or tints for emphasis, one made use of the scaling of visual elements to draw attention to important aspects, and six made use of a change in the look and feel of lines to draw the readers’ attention to a specific detail. It is important to note that more than one use of visual emphasis can be used in one data visualisation. On *Daily Maverick*, 22 data visualisations made use of some visual emphasis– 22 by effects to text, two by changing colour tones or tints, one by scaling visual elements, and two by changing the look and feel of lines in the data visualisation. Only one of the data visualisations on *CNN Digital* did not make use of visual emphasis, meaning that 22 did. The breakdown is that 21 used an effect to text for emphasis, three used changes in colour tones and tints, two used the scaling of visual elements, and a further two made us of a change in the look and feel of lines. In stories from *The New York Times*, 56 data visualisations made use of visual emphasis. Of these, 55 made use of some effects to text used, 14 used changes in colour tones and tints, two made use of the scaling of visual elements, and 21 used a change in the look and feel of lines for visual emphasis.

**Fig 12:**
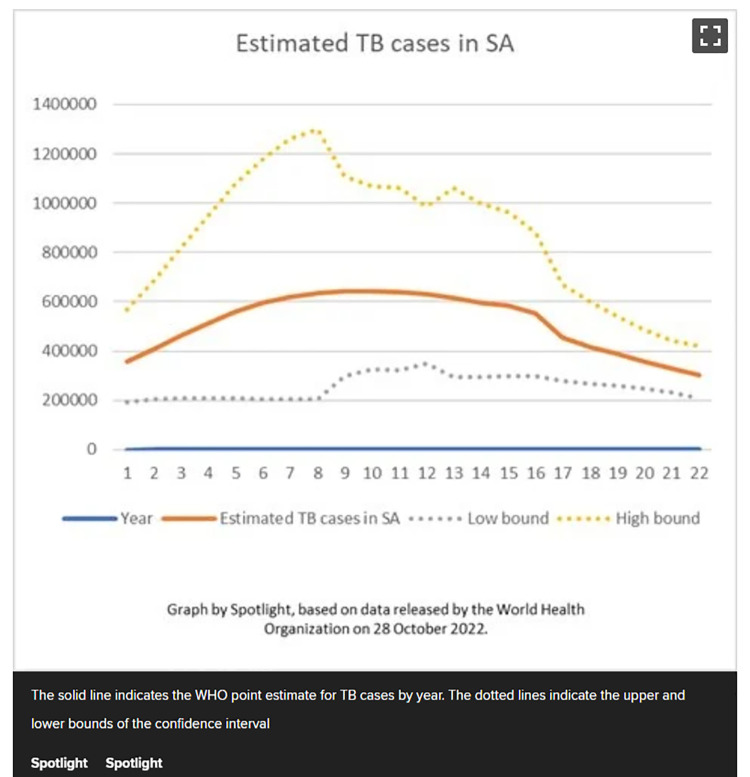
*News24*, ‘Estimated TB cases in SA’, published on 31 October 2022.

In Fig 12, the different line styles focus the reader’s attention on the central line in bold and orange, thereby reducing the cognitive load required to process this data visualisation and thus helping the reader to grasp it more quickly and effectively [2].

**Fig 13:**
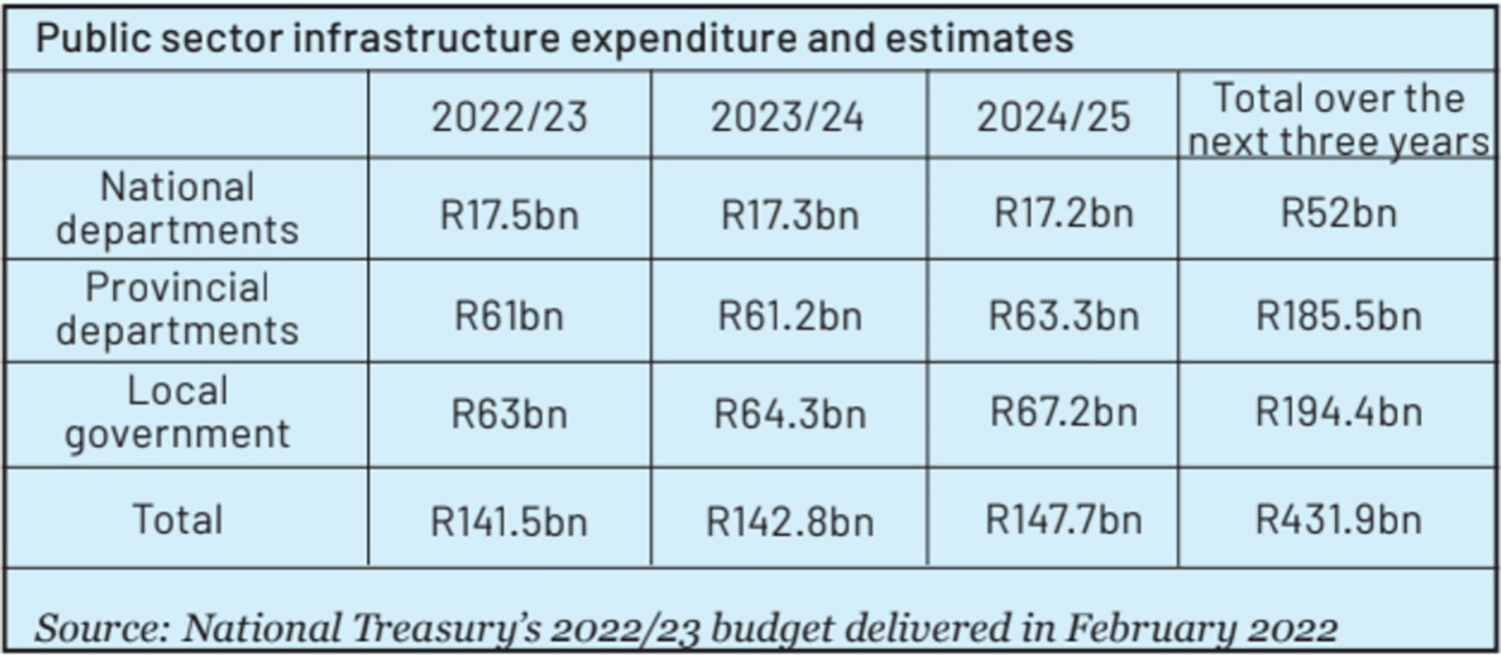
*Daily Maverick*, ‘Ramaphosa’s mega infrastructure roll-out plan failing to fly’, published on 30 October 2022.

The table in Fig 13 is an example where the use of visual emphasis could have helped to make this data story more effective. This data visualisation uses single colour that in itself allows for limited contrast and emphasis [26], especially when the entire table is filled with colour. According to Knaflic [2], colour should be used intentionally and “there needs to be sufficient contrast to make something draw your audience’s attention”. However, this application of colour across all cells of the table does not allow for emphasis, and instead of details of the data visualisation being highlighted to the reader, the cognitive load is quite high, and the meaning of the data visualisation is not immediately clear. Visual emphasis, even by means of filling only select cells with colour and selectively adding bolded text, could have helped to make the meaning of this data visualisation clearer.

### Theme 7: Purpose and intent of the visualisation in the news story

Below, we elaborate on the three aspects of this analysis: a) the topics of the science news stories, b) the purpose or intent of the data visualisations in the news stories, c) the kind of data stories that the data visualisations told in the context of the news story.

#### a. Themes of the science news stories

**Fig 14:**
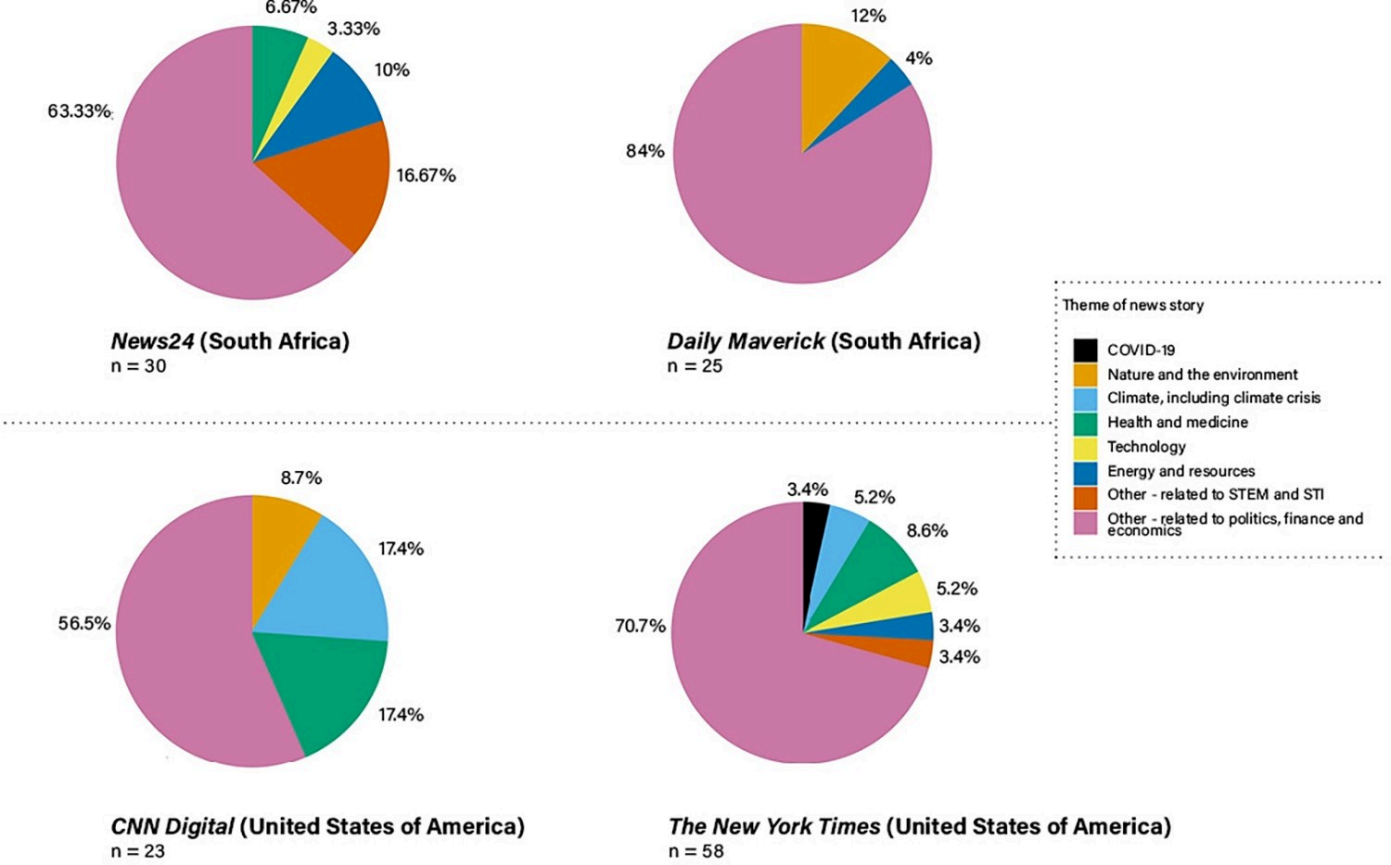
Themes of the science news stories per platform (colour scheme selected for increased reader accessibility).

There were notable differences in the distribution of story themes across the four platforms within and between countries (Fig 14). For example, only *Daily Maverick* and *CNN Digital* had stories about nature and the environment (12% and 8.7% respectively) and only *CNN Digital* and *The New York Times* had stories about climate (17.4% and 5.2% respectively). Notably, only *The New York Times* published any Covid-19-related news stories during October 2022. This is an indication that this theme no longer dominated news agendas, as was the case during 2020 [35, 36].

#### b. The purpose or intent of data visualisation in the context of the news story

Our findings show that, across all platforms, data visualisations were most frequently used to illustrate changes in systems over time, with all four news media platforms featuring data visualisations with this purpose most frequently–*CNN Digital* shares this with ‘enable a deeper understanding of a phenomenon’ (indicated with grey shading in Table 4). We found examples of all the other types of purpose and intent from the theoretical typology in our dataset.

**Table 4:** Purpose or intent of the data visualisation used per platform.

Purpose or intent of data visualisation used	Count per platform			
	South Africa		United States of America	
	*News24* *[n = 30]*	*Daily Maverick* *[n = 25]*	*CNN Digital* *[n = 23]*	*The New York Times* *[n = 58]*
Refute claims		4	2	2
Reveal unintended consequences		1	2	4
Reveal information of personal interest	8	5	3	3
Enable a deeper understanding of a phenomenon	4	5	6	12
Reveal anomalies and deficiencies in systems	5	3	1	3
Track changes in systems	11	7	6	25
Reveal information about an entity in increasing levels of details			2	9
None of the above	2		1	

**Fig 15:**
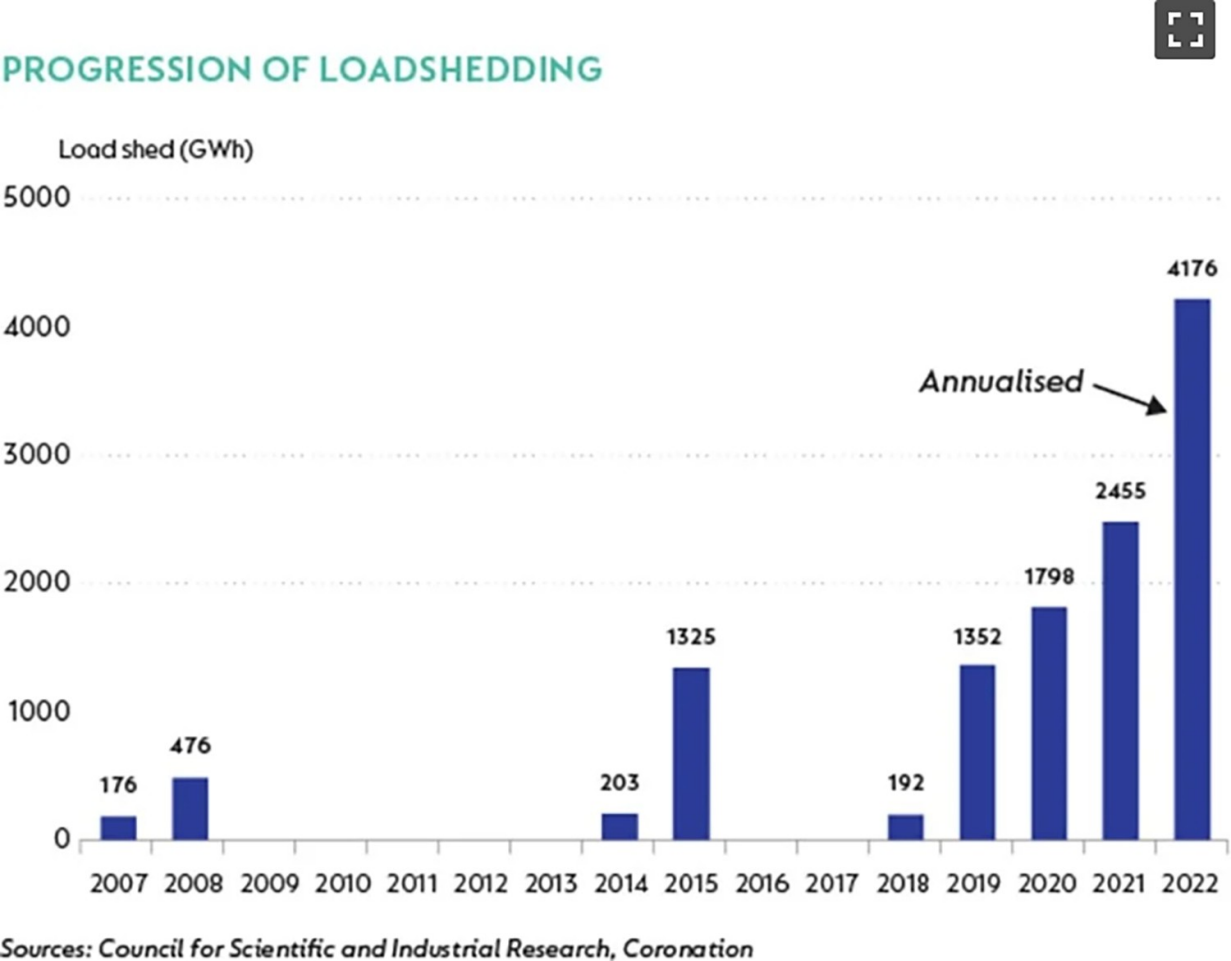
*News24*, ‘Progression of loadshedding’, published on 3 October 2022.

**Fig 16:**
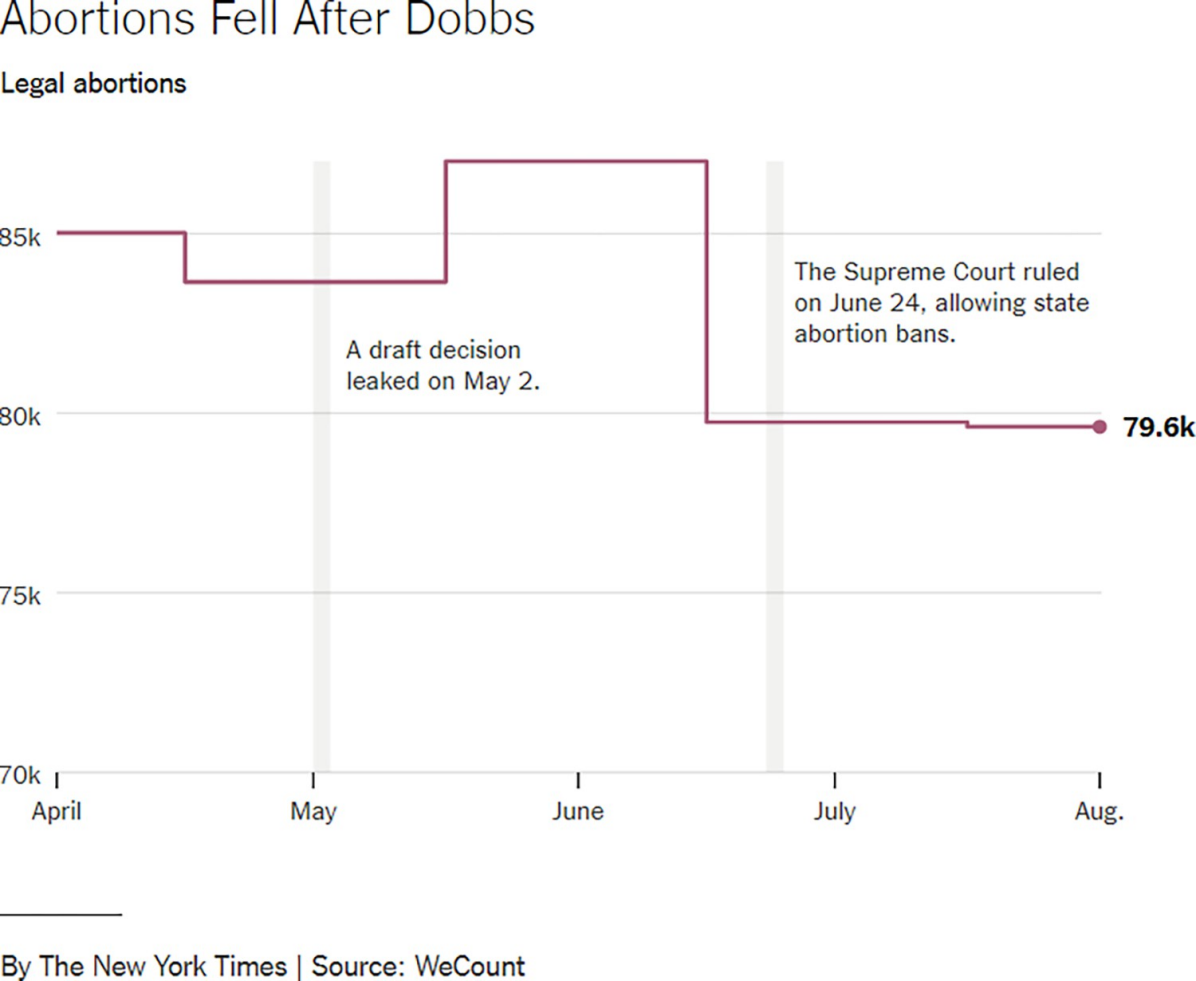
*The New York Times*, ‘Abortions fell after Dobbs’, published on 30 October 2022.

Figs 15 and 16 are examples of data visualisations with the purpose or intent of tracking changes in systems. Fig 15 is from *News24* and shows the progression of loadshedding (scheduled electricity cuts) since it was implemented in South Africa by Eskom in 2007. The theme of this data visualisation is ‘energy and resources’, and a simple bar chart is used to communicate these changes over time. Additional text and simple visual elements (arrows) help the reader to make sense of the data visualisation.

Fig 16, from *The New York Times*, makes use of a stepped line graph to communicate the changes in the number of legal abortions in the US along changes in legislation. These changes in legislation are highlighted to the reader by using additional text and bands of grey to mark moments in time, as indicated on the x-axis. The theme of this visualisation is ‘health and medicine’.

#### c. The kind of data story told by the data visualization

Our findings show that, across all platforms, the most frequently told data stories were those showing changes over time and those comparing two or more discreet values. The highest values are shaded in grey in Table 5. Our dataset showed no examples of data stories used to show hierarchies, browse large databases, or envision alternate outcomes. The data stories that were coded as ‘none of the above’, included data visualisations that where either closely tied to Table 4‘s purpose of revealing information about a matter in increasing levels of details (not adequately covered by Cohen), or did not really tell a successful data story to begin with and therefore the kind of story told could not easily be identified.

**Table 5:** Kind of data story told by the data visualisation, per platform.

Kind of data story told by the data visualisation	Count per platform			
	South Africa		United States of America	
	*News24* *[n = 30]*	*Daily Maverick* *[n = 25]*	*CNN Digital* *[n = 23]*	*The New York Times* *[n = 58]*
Showing changes over time	11	9	8	29
Comparing values	13	7	6	21
Highlighting connections between different variables	3	5	4	3
Tracing flows	1	2		3
Showing hierarchy				
Browsing large databases				
Envisioning alternate outcomes				
None of the above	2	2	5	2

**Fig 17:**
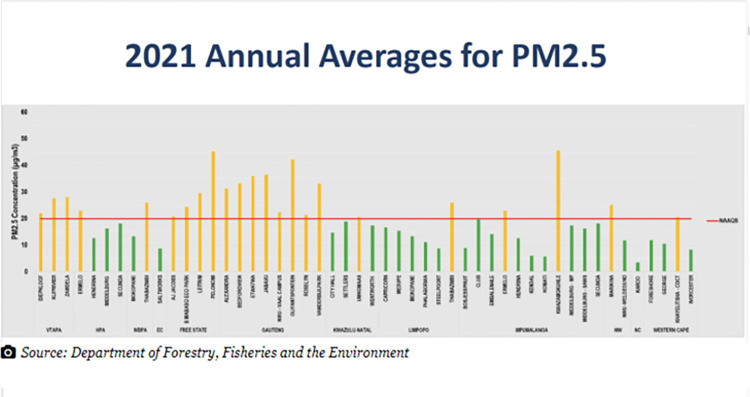
*Daily Maverick*, ‘2021 annual averages for PM2.5’, published on 5 October 2022.

**Fig 18:**
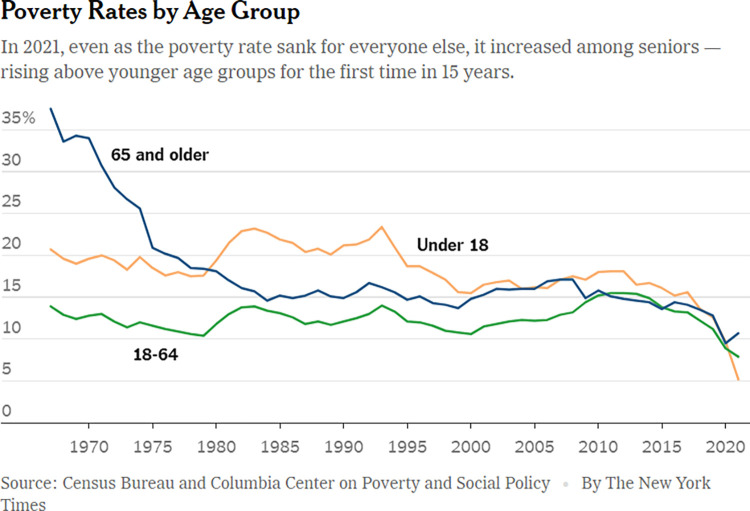
*The New York Times*, ‘An uptick in elder poverty: A blip, or a sign of things to come?’, published on 17 October 2024.

Figs 17 and 18 show examples of data stories that, respectively, compare values and show changes over time. Fig 17, from *Daily Maverick*, is a data visualisation within the theme of ‘nature and the environment’. Unlike the bar graph in Fig 15, this simple bar graph is used to compare discreet values with each other–levels of fine particular matter (PM2.5) as air pollution, across South Africa’s nine provinces. While the use of distinct colours helps the reader to make sense of the data story, the use of background colour and graph lines are distracting, and the storytelling potential of this data visualisation is negatively impacted.

Fig 18, from *The New York Times*, is a simple line graph that draws attention to the changes of poverty rates by age groups in the US, over the course of 50 years. Even though this data visualisation is simple, its use of a distinct qualitative colour scale and bold text helps the reader understand this data story and the changes over time that it presents.

## Limitations of study

Our study was subject to some key limitations that impact the results we attained and the discussions that we were able to have. The first of these relates to the site of production; we did not have access to the motivations and choices of the news platforms, so instead looked at the type of visualisations, as well as the purpose and intent of the data story in the context of the news story as a proxy of these motivations or choices. A future study could consider the motivations and choices of the news platforms, the site of production, more deliberately to add nuance to discussions around the design of data visualisations. Furthermore, we only looked at data visualisations in stories about science, but data visualisations are used in many other articles that work with some form of data and that it would be good to expand on other categories of news stories in future research.

A second limitation relates to the site of audiencing and the exclusion criteria we used to finalise our data set. All examples that contained no colour (i.e., data visualisations in black and white or grayscale) and no text (this includes no annotations, labelling, titles, etc.) were removed from the dataset. We only analysed one visualisation per article, selecting either the main (most prominent) visualisation or the first visualisation in each article. Furthermore, duplicate examples were removed, since most of the coding would be exactly the same and would skew the results. Future studies could use other exclusion criteria, based on a different codebook, and have the opportunity to code data visualisations that do not contain colour or text, and data visualisations that were published om more than one site. This would allow a wider scope of audiencing, as other readers might have different interpretations or reader experiences of the data visualisations that were removed from our data set. Furthermore, data visualisations published on more than one platform might give readers a more nuanced and complete understanding of the data visualisation, since they see it in multiple contexts.

## Conclusions

This study investigated the similarities and differences in the use of data visualisations in science-related news stories via online news platforms in South Africa and the USA, in order to compare the design elements and their relationship to the content of the news stories of the data visualisations on these platforms. Making use of quantitative and qualitative content analysis, we explored the visual and textual elements of data visualisations to understand how these may contribute to or detract from the content and understanding of online science news stories. With this study, we hoped to contribute to the body of knowledge about data visualisations and its links with science communication and science journalism.

### Practical recommendations for data visualisations

There are some key takeaways for science communicators and journalists from this study. First, the use of visual emphasis is encouraged, as this enhances the meaning of a data visualisations. This can be done by highlighting certain elements of the text—indicate titles in bold, draw attention with italics, etc., use tints and tones of colour to draw the viewer’s attention to important details, make use of scaling, and also play with the look and feel of lines to create visual emphasis. Even ‘simple’ graphs, like Figs 11 and 15, can draw the reader’s attention to the most important information, when adequate emphasis is used.

The second takeaway is that labelling specific elements in a data visualisation can help to clarify its meaning. This can help to ensure that the reader understands exactly what each of the elements in the data visualisation is or refers to. This can also be achieved by labelling the relationship between different elements in a visualisation (Fig 6).

Thirdly, make use of a combination of colours, but keep this to a limited range. This study shows that the use of single colour in a data visualisation rarely clarifies the meaning of that visualisation. Instead, it is helpful to use a combination of colours, although not too many, in which the colours help each other make sense within the context of the data story.

The last takeaway from this study is to not shy away from using so-called ‘simple graphs’—the use of overly complicated data visualisations might not land with the reader. The kind of data visualisation used needs to be based on the story the data ought to tell. The purpose or intent of the science communication story, along with its intended audience, should guide the decision regarding the type of visualisation to use. The added value of simplicity in data visualisation is that it allows readers to understand data stories with minimal cognitive load and may be more accessible to audiences with lower ‘visual-numeric literacy’ [20]. This means that readers who are not as ‘graph literate’ and read news stories, including data visualisations, at a glance, have a higher chance of understanding and interpreting the intended meaning of the data visualisation and the news story.

## Suggestions for further research

In a 2023 study, Tong examined “audience perception of the trustworthiness of Covid-19 data visualisations in UK newspaper coverage” [37]. While our study focused on the site of the visual itself [13], future research could relate to previous work by Wijnker et al. [29] more explicitly, to extend this focus to also consider the ways in which readers of *Daily Maverick*, *News24*, *CNN Digital* and *The New York Times* understand and make sense of the data visualisations used on these platforms.

Heravi and Lorez [38] focus on data journalism practices to understand more about global “data journalism-related practices in newsrooms” [38]. In a similar vein, follow-up studies to this current one may focus on the site of production [13], to investigate newsroom practices and production choices of *Daily Maverick*, *News24*, *CNN Digital* and *The New York Times* behind making use of data visualisations in science news stories.
